# Estimating seasonal variation in Australian pertussis notifications from 1991 to 2016: evidence of spring to summer peaks

**DOI:** 10.1017/S0950268818003680

**Published:** 2019-03-20

**Authors:** R. N. F. Leong, J. G. Wood, R. M. Turner, A. T. Newall

**Affiliations:** 1 School of Public Health and Community Medicine, University of New South Wales, Sydney, New South Wales, Australia; 2 Biostatistics Unit, Dean's Office Dunedin School of Medicine, University of Otago, Dunedin, New Zealand

**Keywords:** Australia, notifications, pertussis, seasons, time-series, whooping cough

## Abstract

Unlike for many other respiratory infections, the seasonality of pertussis is not well understood. While evidence of seasonal fluctuations in pertussis incidence has been noted in some countries, there have been conflicting findings including in the context of Australia. We investigated this issue by analysing the seasonality of pertussis notifications in Australia using monthly data from January 1991 to December 2016. Data were made available for all states and territories in Australia except for the Australian Capital Territory and were stratified into age groups. Using a time-series decomposition approach, we formulated a generalised additive model where seasonality is expressed using cosinor terms to estimate the amplitude and peak timing of pertussis notifications in Australia. We also compared these characteristics across different jurisdictions and age groups. We found evidence that pertussis notifications exhibit seasonality, with peaks observed during the spring and summer months (November–January) in Australia and across different states and territories. During peak months, notifications are expected to increase by about 15% compared with the yearly average. Peak notifications for children <5 years occurred 1–2 months later than the general population, which provides support to the theory that older household members remain an important source of pertussis infection for younger children. In addition, our results provide a more comprehensive spatial picture of seasonality in Australia, a feature lacking in previous studies. Finally, our findings suggest that seasonal forcing may be useful to consider in future population transmission models of pertussis.

## Introduction

Pertussis, or whooping cough, is an acute upper respiratory infection of humans primarily caused by *Bordetella pertussis* bacterium. Transmission occurs via airborne droplets expelled from coughing- or sneezing-infected individuals. Despite high vaccination coverage and very large reductions in incidence of serious disease, pertussis infection remains endemic in most settings, including Australia [1].

Pertussis epidemics are known to cycle with peaks occurring every 3–5 years [1, 2]. This pattern has been attributed to the replenishment of the susceptible population fraction due to births, waning of immunity or mortality amongst the immune population, leading to cyclic changes in incidence in endemic settings [1, 3]. Unlike other respiratory infections, there is a lack of consensus around the nature and extent of pertussis seasonality. While studies have suggested that pertussis exhibits seasonality, there remains debate as to the timing of infection peaks and the causes of these seasonal fluctuations. For instance, evidence of seasonal fluctuations in pertussis incidence in some countries has suggested increases during the spring–summer months [4–8]. However, there are also studies which, through exploring relationships between notifications and environmental factors, have suggested pertussis may be more evident during winter months [9, 10]. The US Centers for Disease Control and Prevention has stated that pertussis has no distinct seasonal pattern, but acknowledged that the infection may increase during summer–fall [11]. As with many respiratory infections, pertussis transmission is thought to be influenced by seasonal variations in the environment, human behaviours and to some extent host immune responses, potentially leading to seasonal oscillations in incidence [7, 12–14].

In Australia, pertussis is a nationally notifiable disease with laws mandating that both confirmed and probable cases of the infection must be reported to the Commonwealth's National Notifiable Diseases Surveillance System (NNDSS) [2]. Among vaccine-preventable diseases of childhood, pertussis is the most frequently notified, with Australia reporting the highest rates of notifications amongst high-income countries since the early 1990s [2, 15]. There have been many proposed reasons for the increase in reported incidence of pertussis in Australia, including increased and more sensitive testing and declining population immunity [1, 16, 17].

Multiple epidemics of pertussis occurred in Australia beginning 2000s with varying timing and frequency across different states and territories [2, 18]. Descriptive analyses of notification trends suggest that epidemic peaks occur every 3–4 years [2] and that monthly notifications increase from late winter to early summer in Australia [19]. A recent Australian study evaluated both notification counts and proportions of tests positive for pertussis via polymerase chain reaction (PCR) and serological tests from 2008 to 2011 in Queensland (QLD), Australia, finding a peak in the positive test proportion from October to February (spring to summer) [6]. This contrasts, however, with another QLD study using spatio-temporal analysis of monthly notifications and meteorological data for pertussis, which found a negative association between temperature and pertussis cases suggesting increased winter transmission [10].

This article aims to characterise seasonal variations in incidence of notification rates in Australia from a statistical perspective with the use of time-series models, which have been extensively used to study infectious diseases surveillance data. Given varying climatic conditions across Australian states and territories, we also aim to determine if seasonal patterns vary by state and territory. Finally, as pertussis infection burden is very different across all age groups, we examine seasonality by age group.

## Methods

### Data and study setting

We obtained monthly counts of pertussis notifications in Australia from January 1991 until December 2016 from the NNDSS (see Acknowledgements) with additional stratification by age group and jurisdiction. In Australia, both confirmed and probable cases are notifiable. Confirmed cases must have either a laboratory-definitive evidence (isolation of *B. pertussis* from a clinical specimen, detection by nucleic acid testing or seroconversion in paired sera tests for *B. pertussis* using whole cell or specific antigen/s in the absence of recent vaccination) or a combination of both laboratory-suggestive (significant change in antibody level to *B. pertussis* whole cell of antigen/s, or single high IgG and/or IgA titre to pertussis toxin, or single high IgA titre to whole cell *B. pertussis* antigen, all in the absence of recent vaccination) and clinical evidence (a coughing illness of ⩾2 weeks or specific whooping cough symptoms such as paroxysms of coughing an inspiratory whoop or post-tussive vomiting) [20]. Probable cases are those which have both clinical and epidemiological evidence (an illness 6–20 days after contact with a person likely to be infectious with pertussis and at least one case in the chain of epidemiologically linked cases having confirmatory or suggestive laboratory evidence) [20]. For a complete description of the case definition, see [20]. These definitions are standardised across states and territories in Australia [2].

Data were made available for all states and territories except for the Australian Capital Territory (ACT). We requested data that combined some jurisdictions due to small cell counts, with the analytic regions provided as follows: (1) New South Wales (NSW); (2) Victoria and Tasmania (VIC/TAS); (3) QLD and Northern Territories; (4) South Australia (SA); and (5) Western Australia (WA). Count data were also stratified into the age groups <6 months, 6 months to 4 years, 5–9, 10–64 and ⩾65 years, with the width of these groups reflecting the changing disease rates with age [18] and potential vaccination strategies [21].

### Statistical analysis

Monthly pertussis notifications were standardised to a 30-day month, i.e.

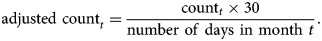


Prior to fitting the full model, we first identified the appropriate multiannual cycle lengths in the overall and state/age notification series via inspection of peaks in the periodograms generated by applying the Fast Fourier Transform.

A time-series model applying the decomposition method, previously applied to other respiratory pathogens [22, 23], was used to extract information on seasonality while accounting for other components (e.g. trends). Such models are useful in this context because they can provide robust estimates of seasonal effects while controlling for other known factors such as changes due to epidemic cycles and change points [24]. In this regard, we formulated a generalised additive model (GAM) to characterise seasonal patterns in pertussis notifications (counts per month), which offered two advantages for our purpose: (1) flexibility to specify the distribution from which our series is generated; and (2) the ability to fit polynomial-based spline functions to complex trend patterns. A quasi-Poisson log-link function was used to account for overdispersion. A change point was also included to account for observed changes in both mean and variance late in the series. In addition, we tested for autocorrelation (AR) in the series to decide if the model needs to include AR terms.

The model was formulated as:

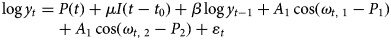
where

*y*_*t*_: =monthly-adjusted pertussis notifications for month *t*

*P*(*t*): =smoothed polynomial-order splines trend component at time *t*

*μI*(*t* − *t*_0_): =shift in the mean where *I*(*t* − *t*_0_) is the Heaviside unit-step function, *t*_0_ is the change point and *μ* is the magnitude of the shift

*β*log *y*_*t*−1_: =autoregressive component of lag order 1

*A*_1_cos (*ω*_*t*,1_ − *P*_1_): =cosinor corresponding to monthly effects

*A*_2_cos (*ω*_*t*,2_ − *P*_2_): =cosinor corresponding to multiannual cycle

*ω*_*t*,*s*_: = 2*π*(*t* − 1/*D*_*s*_), where *D*_1_ = 12 and *D*_2_ is the multiannual cycle length in months determined from the periodogram for the relevant notification series

ε_*t*_: =model random error assumed to follow a white noise process with *t* = 1, 2, …, 312 as indices for the months beginning January 1991 up to December 2016.

Both annual and multiannual seasonal components were formulated as full-cycle cosinor terms (i.e. the seasonal cycle can be explained by sines and cosines) in the model. We tested inclusion of higher frequency cosinor terms but did not find substantial improvements in model fit or changes in estimates of seasonal components. For each cosinor component, *A* corresponds to the amplitude of the cycle and *P* (the phase) determines the timing of peaks for each cycle. In our model, we assumed stationary seasonal effects, i.e. the intra- and inter-annual seasonal effects do not change with time. Further technical details are provided in Supplementary Material S1.

We also used a long time series (spanning 26 years) in order to have more statistical power in determining seasonal and multiannual components. We report peaks in terms of calendar months based on the estimated phase of the cosinor component. In addition, we specify this more accurately using a month fraction reported as either early, mid or late. Since this is a Poisson-type model, the yearly amplitude is interpreted as the % increase observed in notifications during the peak month as compared with the yearly average. Estimates of the multiannual period and amplitude are also reported for completeness.

All analyses were conducted in R, with individual models fitted using the *gam* function of the *‘mgcv*’ package [25]. Confidence intervals (CI) were estimated using the *δ*-method. Goodness-of-fit was assessed using the model deviance explained (*D*^2^) metric.

### Sensitivity analysis

In addition, we conducted sensitivity analyses investigating the interaction between the changepoint and the multiannual cycle lengths and how seasonal and multiannualcomponent estimates changed when removing the AR term from the model.

### Ethical approval

Ethical approval for this research was provided by the UNSW Human Research Ethics Advisory Panel (approval number HC17623).

## Results

### Descriptive summary

The data consisted of 306 147 pertussis notifications between January 1991 and December 2016. The monthly mean was 981 notifications but this masked a strong increasing trend, subject to large fluctuations, with the mean rising from 376 notifications/month during the 1990s through 875 notifications/month during the 2000s to 1910 notifications/month during the period 2010–16.

### Identification of other model components

Estimates of the multiannual cycle length based on the periodograms are provided in Table 1. In general, the multiannual cycle length of pertussis was just below 4 years, with the exception for VIC and TAS (3.0 years) where it was shorter, and SA (4.4 years) and the ⩾65 years old age group (5.3 years) which had longer cycles. Despite these differences, the estimated values are reasonably consistent with previous Australian literature that identifies epidemic cycles of around 3–4 years [2].

**Table 1.** Summary of key seasonal signatures of monthly pertussis notifications from January 1991 to December 2016 for all of Australia and for each subgroup (by states and territories and by age groups)

	Multiannual cycle length in years	Peak month	Yearly amplitude	Multiannual amplitude
*Australia*	3.8 years	Early November (10.80–11.78)	1.15 (1.11–1.19)	1.04 (1.01–1.09)
States/Territories				
*New South Wales*	3.8 years	Early November (10.45–11.74)	1.15 (1.10–1.20)	1.05 (1.00–1.10)
*Victoria and Tasmania*	3.0 years	Mid-December (11.57–1.22)	1.13 (1.07–1.19)	1.08 (1.02–1.13)
*Queensland and Northern Territory*	3.8 years	Early December (11.40–12.87)	1.13 (1.08–1.18)	1.06 (1.01–1.12)
*South Australia*	4.4 years	Early October (9.58–10.92)	1.18 (1.11–1.24)	1.06 (1.00–1.12)
*Western Australia*	3.8 years	Mid-November (10.84–12.43)	1.17 (1.10–1.25)	1.06 (0.99–1.13)
Age groups				
*<6 months*	3.8 years	Mid-January (12.65–2.07)	1.20 (1.12–1.27)	1.15 (1.07–1.23)
*6 months – 4 years*	3.8 years	Early January (12.49–1.92)	1.16 (1.10–1.22)	1.06 (1.00–1.11)
*5 years – 9 years*	3.8 years	Mid-November (10.85–11.90)	1.22 (1.16–1.28)	1.05 (1.00–1.10)
*10 years – 64 years*	3.8 years	Late October (10.46–11.41)	1.15 (1.11–1.19)	1.04 (1.01–1.08)
⩾*65 years*	5.3 years	Late November (11.00–12.58)	1.15 (1.09–1.21)	1.13 (1.05–1.21)

Numbers inside ( ) are the estimated 95% CIs. Numbers corresponding to the peak month are interpreted as the month and its part in a 12-month calendar. Amplitude is interpreted as the % increase observed in notifications during the peaks as compared to the seasonal average.

The change point analysis found structural breaks in July 2008 in all series, except for WA where the break occurred in June 2004. We found that both the mean and variance of each series increased at the change points.

### Model fit

**Fig. 1.**
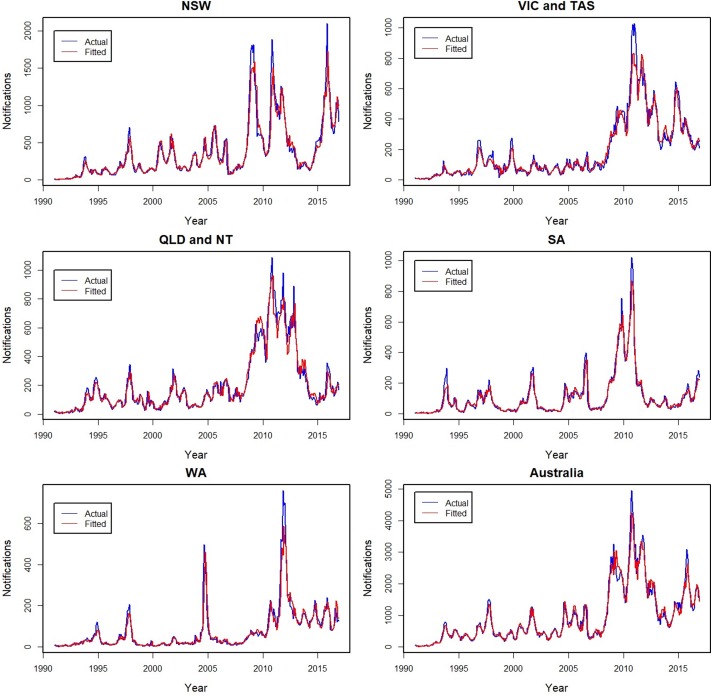
Actual (blue −) and fitted (red −) monthly pertussis notifications from January 1991 to December 2016 by states and territories and for all of Australia (from upper left: New South Wales (NSW), Victoria and Tasmania (VIC and TAS), Queensland and Northern Territory (QLD and NT), South Australia (SA), Western Australia (WA) and Australia).

**Fig. 2.**
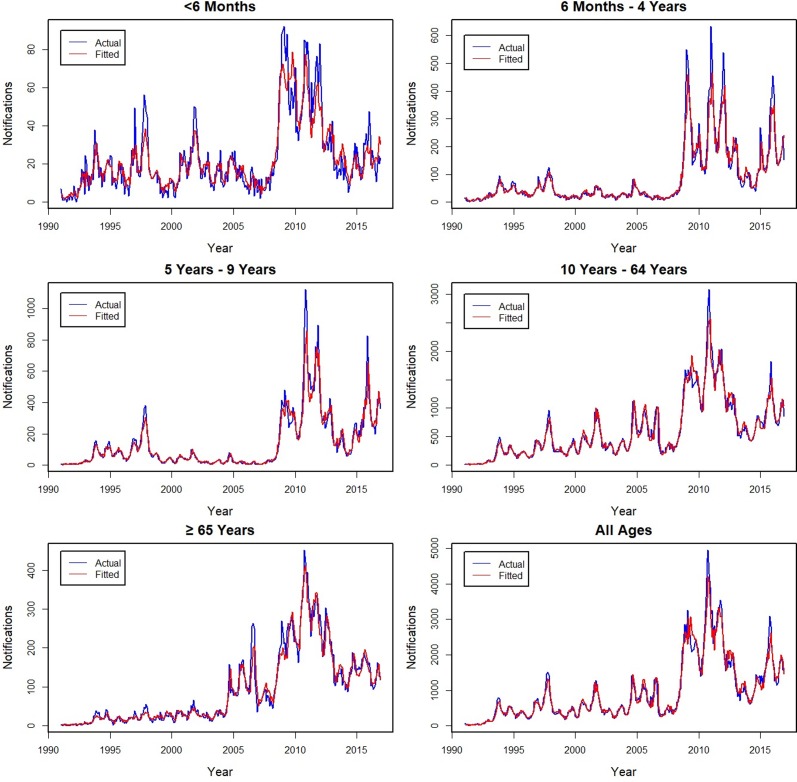
Actual (blue −) and fitted (red −) monthly pertussis notifications from January 1991 to December 2016 by age groups and for all ages (from upper left: <6 months, 6 months to 4 years, 5–9, 10–64, ⩾65 years and all ages).

The actual and fitted monthly notifications are shown for Australia and by state and territory (Fig. 1), with the age group fits shown in Figure 2. The models fit well for all series, with individual *D*^2^ values ranging from 90% to 97% for all subgroups, except for the youngest age group for which it was lower at 79%. Errors in the fit were primarily related to underestimating peaks, with younger age groups for which peaks were more common having lower *D*^2^ values. The influence of peaks is especially apparent during the year 2010 for all series.

The fitted trend and shift components are provided in Supplementary Material S2. Notification series in other states were estimated to increase by >20% from 2008 with the exception of WA. For these series, change appeared to be driven by rises in notifications in children aged <5 years (around 40–50%) with relatively small changes observed for ⩾65-year olds (around 10%). In the case of WA, an earlier change point was found (2004) but the scale of change was minimal (≈3%).

### Seasonal characteristics of pertussis notifications

**Fig. 3.**
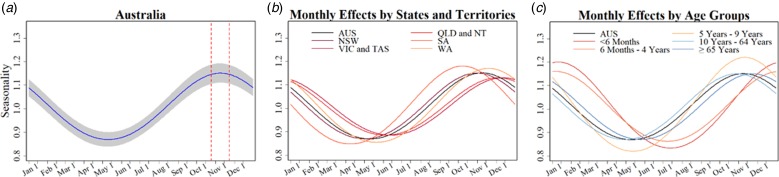
(From left to right) (a) Estimated monthly % changes in amplitude for the general Australian population (shading indicates 95% CI) and dashed lines delineate 95% CI for peak month. (b) Estimated monthly changes in % by states and territories (colours as shown in legend) and for all of Australia (black). Abbreviations: New South Wales (NSW), Victoria and Tasmania (VIC and TAS), Queensland and Northern Territory (QLD and NT), South Australia (SA), Western Australia (WA), and (AUS) Australia. (c) Estimated monthly changes in % by age groups (colours as shown in legend) and for all of Australia (black).

Table 1 summarises key seasonal characteristics of pertussis notifications for the whole of Australia and for each subgroup as estimated by the model. Figure 3 provides visual representations of the fitted seasonal components for each series. For Australia as a whole, we estimated that pertussis notifications peak during early November, with the peak notifications around 15% higher than the yearly average. The amplitude and timing of peaks was similar in most states and territories with the exception of an earlier peak in SA (early October), and later peaks in VIC and TAS (late December to early January).

In respect to the age-group models, we found larger peak-and-trough variations of ≈20% in two of the younger age groups, <6 months and 5–9 year olds, compared with ≈15% in the older age groups. Peak months in those children <5 years were similar but were delayed by almost 2 months.

### Sensitivity analysis

Multiannual cycle lengths based on the entire series and the data prior to the identified change points are provided separately in Supplementary Material S3 Table S1. In most cases, this led to a minor decrease in cycle lengths (<2 months), but for WA, it rose from 46 to 54 months, while for the ⩾65-year groups, it fell from 64 to 54 months. For these larger changes, we refitted the models with adjusted multiannual cycle lengths but found no changes in seasonal estimates, while the absolute changes in amplitudes for multiannual estimates were smaller by <0.03.

Results from the models without the autocorrelation term are provided in Supplementary Material S3 Table S2 and Figures S3 and S4. Model fits were much poorer (*D*^2^ dropped by at least 5% to as much as 15%). However, we still found strong evidence of seasonal and multiannual effects with increased amplitudes. In the case of the national data, yearly amplitude increased from 1.15 to 1.30, multiannual amplitude increased from 1.04 to 1.31, and peak month has shifted from November to January. These suggest that there are some identifiability issues in regard to the multiannual, seasonal and AR(1) components. Nevertheless, while model estimates were sensitive to the removal of the AR(1) component, the relative timing and relative amplitude sizes among subgroups were similar to the results of the original model.

## Discussion

This article describes the seasonal characteristics of pertussis notifications in Australia as observed from 1991 to 2016 using a time-series decomposition approach. We found that pertussis notifications in Australia exhibit seasonal variation with peaks beginning from early November in the overall population but with delays until January and February for younger children. These findings add to previous descriptive studies that have noted seasonality in pertussis in Australia by establishing more robust estimates of its phase and amplitude. The study provides an important additional insight, evaluating in detail how timing and seasonal amplitude varied by geographic locations and age groups, which were not analysed in the previous Australian studies [6, 10] or in several studies for other countries [4, 7, 8].

Our findings were largely in line with the previous Australian results of Kaczmarek *et al*. [6] which examined the proportion of positive tests (PCR and serology) for pertussis from 2008 to 2011 in the state of QLD. Based on descriptive statistics, they found peaks during October to February (spring to summer) for the proportion of tests positive. However, they also examined seasonality in notifications for the same time period but were not able to establish a clear seasonal pattern in notification count data. For our analysis, having a longer data series (26 *vs.* 4 years) and a model which was able to account for long-term trends, epidemic cycles and a change point allowed us to establish a clear seasonal pattern for pertussis using notification counts alone. The peak notifications we found during spring to summer months were also consistent with observations in some other countries, particularly Canada and The Netherlands [4, 7], but were almost a season earlier compared with the USA (summer to fall) [11].

Our results did contrast with another Australian study on QLD [10] which observed that pertussis counts were negatively associated with monthly minimum mean temperature, thus suggesting that pertussis infection must be more prevalent during colder seasons. The study utilised a spatio-temporal statistical model; however, they did not appear to first de-seasonalise pertussis counts and temperature. This could have potentially led to inherent seasonality serving as a confounding variable and a risk of a spurious association between these two series.

We also found new evidence in support of seasonal patterns differing by age group in Australia. It has been hypothesised that age-specific seasonality in infectious diseases is driven by the seasonality of contact patterns [26] and age-based activities such as opening of schools [7, 10]. We found that for children <5 years of age, the peak months were observed 1–2 months later than the general population. The delay in peak timing for these children suggests that pertussis epidemics in Australia could be driven by growth in cases in older children and adults resulting from more activities and social interactions among these age groups [4], with a delayed rise in younger children who have less community contacts. It is also consistent with evidence that shows younger children tend to catch the infection from their older household members [27–29]. Our finding is broadly consistent with the results from British Columbia, Canada which noted that pertussis incidence among adolescents peaked in June which is earlier than peaks for younger children [4]. Likewise results from The Netherlands study which found bimodal summer peaks reflecting the impact of school holidays found the first (July) peak in incidence rates among 13–18 years old [7] more than a month before the peak in infants.

We found that pertussis notifications in Australia have multiannual cycles of approximately 4 years in length, consistent with interepidemic periods observed in pertussis incidence in many countries [1, 7, 30]. We note however that our estimated amplitudes were small. We suspect that this may reflect the autocorrelation term in our model absorbing some of the amplitude of the multiannual cycles. As evidence of this, when we removed the AR(1) term, the multiannual amplitude increased from 4% to 30%, although the model fits to the data were inferior. The multiannual period of 4 years appears to be longer than pre-vaccination cycles in Australia [21, 31] and this agrees with observations from Broutin *et al*. based on data from 64 countries, showing an increase in the interval between epidemics (of 1.3 years) after introduction of vaccination [3].

We note that there appears to be some differences in multiannual cycle lengths across states and territories in Australia. However, since cycle length was a model input, we were not able to assess the statistical significance of these differences across jurisdictions. Nevertheless, these findings provide evidence of geographic differences in the epidemiology of pertussis across Australia [32]. Previous studies have identified smaller population and/or geographic isolation as possible explanations of differences in multiannual cycle lengths [32] (see Supplementary Material S3 Table S1). In addition, some of these differences may be potentially related to variation in vaccination schedule between states with some states funding additional doses during this period beyond the nationally funded National Immunisation Programme (NIP) [2]. For example, NSW funded maternal vaccination during the third trimester of pregnancy in March 2015, before when the strategy was included in the NIP (July 2018).

We estimated a sharp increase in average monthly notifications of about 18% starting in 2008, coinciding with the start of the largest epidemic of pertussis in Australia during our observation period, with a peak of 38 732 notified cases in 2011 [2, 18]. Aside from the epidemic, a switch to predominant use of PCR to confirm pertussis in children and increase use of PCR even in adolescents and adults is likely to have contributed to a rise in the proportion of infections that were notified. Reimbursements for PCR testing under the Medicare Benefits Schedule became available beginning late 2005 which could have influenced the behaviour to conduct more tests across Australia [33]. Indeed, it has been noted that high levels of laboratory testing inflated Australian pertussis case numbers by identifying more ambulatory cases in children and adults [15]. Put together, the increased availability of PCR testing (which is more sensitive than culture) and better awareness on pertussis infection have led to increased testing over time, and may have led to a sustained increase in notification rates [34, 35]. In terms of the epidemic itself, we note that in late 2003, routine pertussis immunisation in Australia saw the introduction of an adolescent booster dose and simultaneous removal of an 18-month booster dose [36]. Dynamic models of the impact of this change suggested a short-term rise in herd immunity, before a larger pertussis epidemic [30] at around the time the real epidemic occurred.

## Limitations

There are several limitations in the analysis presented here and the data on which it was based. The routinely collected data requested did not distinguish between test type that resulted in notification. This is important as different testing patterns within a year are observed for PCR and serology tests [6], the tests differ in diagnostic performance [37] and time trends in testing have occurred, notably the switch to more widespread PCR use [35]. Notification data are also known to underestimate the true incidence of pertussis in Australia, particularly in older age groups [38]. These data represent only a proportion of all cases which have occurred due to under-reporting, representing only those for which health care was sought and the case definition was met (e.g. a laboratory test was conducted). The degree of under-representation is unknown and may vary by jurisdiction due to varying reporting mechanisms and other factors [39]. For example, while Australia has universal healthcare coverage through Medicare, healthcare seekers still incur out-of-pocket expenses which may bias health-seeking behaviour towards those who can access health facilities more readily [40]. However, notification data can still provide useful information on the distribution of cases. Lastly, our model assumed stationary seasonal effects throughout the study period. While this is reasonable to assume with our data, we note the danger of our model not being able to reflect dynamic changes in seasonality resulting from events such as epidemics.

## Conclusion

We found evidence that pertussis exhibits seasonality in Australia notification data. Peaks begin from early November for the overall population but are delayed until January and February for younger children. This supports the theory that older household members remain an important source of pertussis infection for younger children. We illustrate that using a rigorous statistical approach, routinely collected notification count data in aggregated form can provide useful information about the seasonality of pertussis. The pertussis seasonality we have identified suggests that it may be useful to consider seasonal forcing in future population transmission models of pertussis. Further research is needed to identify the drivers of the seasonal variations in pertussis incidence.

## Supplementary material

For supplementary material accompanying this paper visit https://doi.org/10.1017/S0950268818003680. click here to view supplementary material

## References

[1] Jackson D and Rohani P (2014) Perplexities of pertussis: recent global epidemiological trends and their potential causes. Epidemiology and Infection 142, 672–684. 23324361 10.1017/S0950268812003093 PMC9151176

[2] Australian Technical Advisory Group on Immunisation (ATAGI) (2015) Pertussis In The Australian Immunisation Handbook, 10th Edn Canberra: Australian Government Department of Health, pp. 312–326.

[3] Broutin H (2010) Impact of vaccination and birth rate on the epidemiology of pertussis: a comparative study in 64 countries. Proceedings of the Royal Society of London B: Biological Sciences 277, 3239–3245. 10.1098/rspb.2010.0994 PMC2981935 20534609

[4] Skowronski DM (2002) The changing age and seasonal profile of pertussis in Canada. Journal of Infectious Diseases 185, 1448–1453. 11992280 10.1086/340280

[5] Ghorbani GR (2016) Comparing seasonal pattern of laboratory confirmed cases of pertussis with clinically suspected cases. Osong Public Health and Research Perspectives 7, 131–137. 27169013 10.1016/j.phrp.2016.02.004 PMC4850371

[6] Kaczmarek MC (2015) Pertussis seasonality evident in polymerase chain reaction and serological testing data, Queensland, Australia. Journal of the Pediatric Infectious Diseases Society 5, 214–217. 27199473 10.1093/jpids/piu144

[7] de Greeff SC (2009) Seasonal patterns in time series of pertussis. Epidemiology and Infection 137, 1388–1395. 19327200 10.1017/S0950268809002489

[8] Bhatti MM (2015) Eight-year review of *Bordetella pertussis* testing reveals seasonal pattern in the United States. Journal of the Pediatric Infectious Diseases Society 6, 91–93. 10.1093/jpids/piv079 26621328

[9] Gosai A, Salinger J and Dirks K (2009) Climate and respiratory disease in Auckland, New Zealand. Australian and New Zealand Journal of Public Health 33, 521–526. 20078568 10.1111/j.1753-6405.2009.00447.x

[10] Huang X (2017) Assessing the social and environmental determinants of pertussis epidemics in Queensland, Australia: a Bayesian spatio-temporal analysis. Epidemiology and Infection 145, 1221–1230. 28091337 10.1017/S0950268816003289 PMC9507837

[11] US Centers for Disease Control and Prevention (CDC). (2015) Pertussis In Hamborsky J, Kroger A and Wolfe C (eds), Epidemiology and Prevention of Vaccine-Preventable Diseases, 13th Edn Washington, D.C.: Public Health Foundation, pp. 261–278.

[12] Fisman D (2012) Seasonality of viral infections: mechanisms and unknowns. Clinical Microbiology and Infection 18, 946–954. 22817528 10.1111/j.1469-0691.2012.03968.x

[13] Grassly NC and Fraser C (2006) Seasonal infectious disease epidemiology. Proceedings of the Royal Society of London B: Biological Sciences 273, 2541–2550. 10.1098/rspb.2006.3604 PMC1634916 16959647

[14] Nelson RJ (2004) Seasonal immune function and sickness responses. Trends in Immunology 25, 187–192. 15039045 10.1016/j.it.2004.02.001

[15] McIntyre PB and Nolan TM (2014) Pertussis control: where to now? The Medical Journal of Australia 200, 306–307. 24702074 10.5694/mja14.00234

[16] He Q and Mertsola J (2008) Factors contributing to pertussis resurgence. Future Microbiology 3, 329–339. 18505398 10.2217/17460913.3.3.329

[17] Wood N and McIntyre P (2008) Pertussis: review of epidemiology, diagnosis, management and prevention. Paediatric Respiratory Reviews 9, 201–212. 18694712 10.1016/j.prrv.2008.05.010

[18] Pillsbury A, Quinn HE and McIntyre PB (2014) Australian vaccine -preventable disease epidemiological review series: pertussis, 2006–2012. Commmunicable Diseases Intelligence 38, E179–E194. 10.33321/cdi.2014.38.34 25391404

[19] Chiu C (2010) Vaccine preventable diseases in Australia, 2005 to 2007. Communicable Diseases Intelligence Quarterly Report 34, S1. 21416762 10.33321/cdi.2010.34.48

[20] Australian Government Department of Health (2014) Pertussis case definition. In http://www.health.gov.au/internet/main/publishing.nsf/Content/cda-surveil-nndss-casedefs-cd_pertus.htm.

[21] Campbell PT (2015) Defining long-term drivers of pertussis resurgence, and optimal vaccine control strategies. Vaccine 33, 5794–5800. 26392008 10.1016/j.vaccine.2015.09.025

[22] Poljak Z, Carman S and McEwen B (2014) Assessment of seasonality of influenza in swine using field submissions to a diagnostic laboratory in Ontario between 2007 and 2012. Influenza and Other Respiratory Viruses 8, 482–492. 24725968 10.1111/irv.12248 PMC4181809

[23] Willis MD (2012) Seasonality of tuberculosis in the United States, 1993–2008. Clinical Infectious Diseases 54, 1553–1560. 22474225 10.1093/cid/cis235 PMC4867465

[24] Zhang X (2014) Applications and comparisons of four time series models in epidemiological surveillance data. PLoS ONE 9, e88075. 24505382 10.1371/journal.pone.0088075 PMC3914930

[25] Wood S and Wood MS (2015) Package ‘mgcv’. R package version 1–7.

[26] Nguyen HT and Rohani P (2008) Noise, nonlinearity and seasonality: the epidemics of whooping cough revisited. Journal of The Royal Society Interface 5, 403–413. 10.1098/rsif.2007.1168 PMC2607388 17878136

[27] Biellik RJ (1988) Risk factors for community-and household-acquired pertussis during a large-scale outbreak in central Wisconsin. Journal of Infectious Diseases 157, 1134–1141. 3373018 10.1093/infdis/157.6.1134

[28] McIntyre P and Wood N (2009) Pertussis in early infancy: disease burden and preventive strategies. Current Opinion in Infectious Diseases 22, 215–223. 19395958 10.1097/QCO.0b013e32832b3540

[29] Skoff TH (2015) Sources of infant pertussis infection in the United States. Pediatrics 136, 635–641. 26347437 10.1542/peds.2015-1120

[30] Hethcote HW, Horby P and McIntyre P (2004) Using computer simulations to compare pertussis vaccination strategies in Australia. Vaccine 22, 2181–2191. 15149775 10.1016/j.vaccine.2003.11.053

[31] Hall R (1993) Notifiable diseases surveillance, 1917 to 1991. Commmunicable Diseases Intelligence 17, 226–236.

[32] Quinn HE and McIntyre PB (2007) Pertussis epidemiology in Australia over the decade 1995–2005-trends by region and age group. Communicable Diseases Intelligence Quarterly Report 31, 205. 17724997 10.33321/cdi.2007.31.18

[33] Australian Government Department of Health and Ageing (2011) Review of Australia's Health Sector Response to Pandemic (H1N1) 2009: Lessons Identified. Canberra: Commonwealth of Australia.

[34] Kaczmarek M, Ware R and Lambert S (2016) The contribution of PCR testing to influenza and pertussis notifications in Australia. Epidemiology and Infection 144, 306–314. 26112983 10.1017/S0950268815001004

[35] Kaczmarek MC (2013) Sevenfold rise in likelihood of pertussis test requests in a stable set of Australian general practice encounters, 2000–2011. The Medical Journal of Australia 198, 624–628. 23919712 10.5694/mja13.10044

[36] National Center for Immunisation Research and Surveillance (2017) Significant events in diphtheria, tetanus and pertussis vaccination practice in Australia. In http://www.ncirs.edu.au/assets/provider_resources/history/Diphtheria-tetanus-pertussis-history-March-2017.pdf.

[37] Communicable Diseases Network Australia. Australian national notifiable diseases and case definitions. In http://www.health.gov.au/internet/main/publishing.nsf/Content/cdasurveil-nndss-casedefs-cd_pertus.htm.

[38] Bertilone C, Wallace T and Selvey LA (2014) Finding the ‘who’ in whooping cough: vaccinated siblings are important pertussis sources in infants 6 months of age and under. Communicable Diseases Intelligence Quarterly Report 38, 195–200. 10.33321/cdi.2014.38.35 25391405

[39] Dey A (2016) Summary of national surveillance data on vaccine preventable diseases in Australia, 2008–2011. Communicable Diseases Intelligence Quarterly Report 40, S1–70. 27087017 10.33321/cdi.2016.40.11

[40] Duckett S and Willcox S **(CDC)** (2015) The Australian Health Care System, 5th Edn Oxford, UK: Oxford University Press.

