# Supplementary material for: Estimating seasonal variation in Australian pertussis notifications from 1991 to 2016: evidence of spring to summer peaks
Source: Epidemiol Infect. 2019 Mar 20;147:e155. doi: 10.1017/S0950268818003680 (PMC6518527; doi:10.1017/S0950268818003680)

*At Epidemiology & Infection*

Estimating seasonal variation in Australian pertussis notifications from 1991–2016: evidence of spring to summer peaks

R. N. F. LEONG, J. G. WOOD, R. M. TURNER, A. T. NEWALL

**Supplementary Materials**

**S1: Model Framework and Technical Details**

**S2: Estimated Trend + Shift Components**

**S3: Results from Sensitivity Analysis**

**Supplementary Material S1: Model Framework and Technical Details**

To characterize seasonal patterns in pertussis notifications recorded as counts per month, the model is formulated following a log-additive components framework, i.e.,

$$\log\mathrm{notification}s_{t}=\mathrm{Tren}d_{t}+\mathrm{Seaso}n_{t}+\mathrm{Rando}m_{t},$$

where the trend component consists of changes in the series as a function of time and may consider short-order autocorrelation in the series; the seasonal component consists of both monthly effects and multiannual cycles; and random innovations which are assumed to follow a white noise process.

A generalised additive model approach was used which offered two advantages for our purpose: (1) flexibility to specify the distribution from which our series is generated; and (2) ability to automatically fit polynomial-based spline function for the trend component owing to the seemingly complex trend pattern in the series. Note that the other components included in the model only served as controls to ensure that the seasonal effects were estimated correctly. For this reason, the trend component’s complexity is not explicitly controlled but rather is auto-fitted using an algorithm that finds the best-fitting polynomial function of $t$ given the data considering all the other components in the model.

Initial investigation of the monthly means of pertussis notifications showed that a cosinor model (i.e., the seasonal cycle can be explained by sines and cosines) is sufficient to describe the monthly variations. To simplify the model interpretation, the corresponding multiannual epidemic cycle was also formulated as a cosinor term in the model. Lastly, because of the high degree of autocorrelation in the resulting residuals after initially fitting a model with no autocorrelation component, the model was reformulated to include an autocorrelation term of order 1.

Putting all these together, we have the following model:

$$\log y_{t}=P\left( t \right)+\mu I\left( t-t_{0} \right)+\beta\log y_{t-1}+A_{1}\cos(\omega_{t, 1}-P_{s_{1}})+A_{s_{2}}\cos(\omega_{t, 2}-P_{2})+\varepsilon_{t}$$

where

$y_{t} :=$monthly-adjusted pertussis notifications for month $t$

$P\left( t \right) :=$smoothed polynomial-order splines trend component at time $t$

$\mu I(t-t_{0}) :=$shift in the mean where $I\left( t-t_{0} \right)$ is the Heaviside unit-step function, $t_{0}$ is the changepoint, and $\mu$ is the magnitude of the shift

$\beta\log y_{t-1} :=$autoregressive component of lag order 1

$A_{1}\cos(\omega_{t,1}-P_{1}) :=$cosinor corresponding to monthly effects

$A_{2}\cos(\omega_{t, 2}-P_{2}) :=$cosinor corresponding to multiannual cycle

$\omega_{t,s} := 2\pi\left( \frac{t-1}{D_{s}} \right)$, where $D_{1}=12$ and $D_{2}$ is multiannual cycle length in months determined from the periodogram for the relevant notification series

$\varepsilon_{t} :=$model random error assumed to follow a white noise process

with $t=1, 2, \ldots, 312$ as indices for the months beginning Jan 1991 up to Dec 2016.

For the cosinor components, $A$ corresponds to the amplitude, i.e., the difference between the cycle’s peak and mean levels, and $P$ corresponds to the phase measured in radians, i.e., the peak location in time of the cycle. In our model, we assumed stationary seasonal effects, i.e., the intra- and inter-annual seasonal effects do not change over the years. To make the model linear with each cosinor component, we note that:

$$A_{s}\cos\left( \omega_{t,s}-P_{s} \right)=\nu_{1,s}\cos\omega_{t,s}+\nu_{2,s}\sin\omega_{t,s}$$

with

$A_{s}=\sqrt{\nu_{1,s}^{2}+\nu_{2,s}^{2}}$

$P_{s}=\left\{ \begin{aligned} \arctan\left( \frac{\nu_{2,s}}{\nu_{1,s}} \right) , \nu_{1,s}\geq0 \\ \arctan\left( \frac{\nu_{2,s}}{\nu_{1,s}} \right)-\pi, \nu_{1,s}<0 \mathrm{and} \nu_{2,s}\geq0 \\ \arctan\left( \frac{\nu_{2,s}}{\nu_{1,s}} \right)+\pi, \nu_{1,s}<0 \mathrm{and} \nu_{2,s}<0 \end{aligned} \right.$

$\omega_{t,s}=2\pi\left( \frac{t-1}{k} \right)\times cycles completed in a cycle length k in months$

Since the amplitude is non-linear in both $\theta_{s}$ parameters, confidence intervals were estimated using the delta method using the estimated standard errors of the estimates for $\theta_{s}$ parameters.

The model was fitted using the *gam* function of the *‘mgcv*’ package in R. A quasi-Poisson link function is used to account for the overdispersion in the counts data. Model fit was assessed using the model (proportion of null) deviance explained ($D^{2}$) criterion computed as:

$$D^{2}=\frac{\log L\left( \boldsymbol{y} | {\hat{\boldsymbol{\theta}}}_{s} \right)-\log L\left( \boldsymbol{y} | {\hat{\boldsymbol{\theta}}}_{M} \right)}{\log L\left( \boldsymbol{y} | {\hat{\boldsymbol{\theta}}}_{s} \right)-\log L\left( \boldsymbol{y} | {\hat{\boldsymbol{\theta}}}_{0} \right)}\times100\%$$

where $L\left( \boldsymbol{y} | \hat{\boldsymbol{\theta}} \right)$ is the likelihood function for the observation vector $\boldsymbol{y}$ under the fitted parameter vector $\hat{\boldsymbol{\theta}}$, i.e. ${\hat{\boldsymbol{\theta}}}_{\boldsymbol{s}}$ for the saturated model (wherein each observation is assigned its own parameter so that fitting is exact), ${\hat{\boldsymbol{\theta}}}_{\boldsymbol{M}}$ for the model under consideration, and ${\hat{\boldsymbol{\theta}}}_{\boldsymbol{0}}$ for the model with only an (mean) intercept term. As a goodness-of-fit statistic, we say that model $M$ fits well if $0<D^{2}\to1$. Separate models were fitted for each subgroup analysis (by geographic location and by age group) to perform comparisons of seasonal effects between the different groups.

**Supplementary Material S2: Estimated Trend + Shift Components**

Figure S1: Estimated trend + shift components in % estimated from the model for monthly pertussis notifications from January 1991 - December 2016 by states and territories and for all of Australia (from upper left: New South Wales (NSW), Victoria and Tasmania (VIC and TAS), Queensland and Northern Territory (QLD and NT), South Australia (SA), Western Australia (WA), and Australia). Note that the shift (i.e., changepoint) happened in mid-2008 for all groups except for WA when it was observed in mid-2004.**
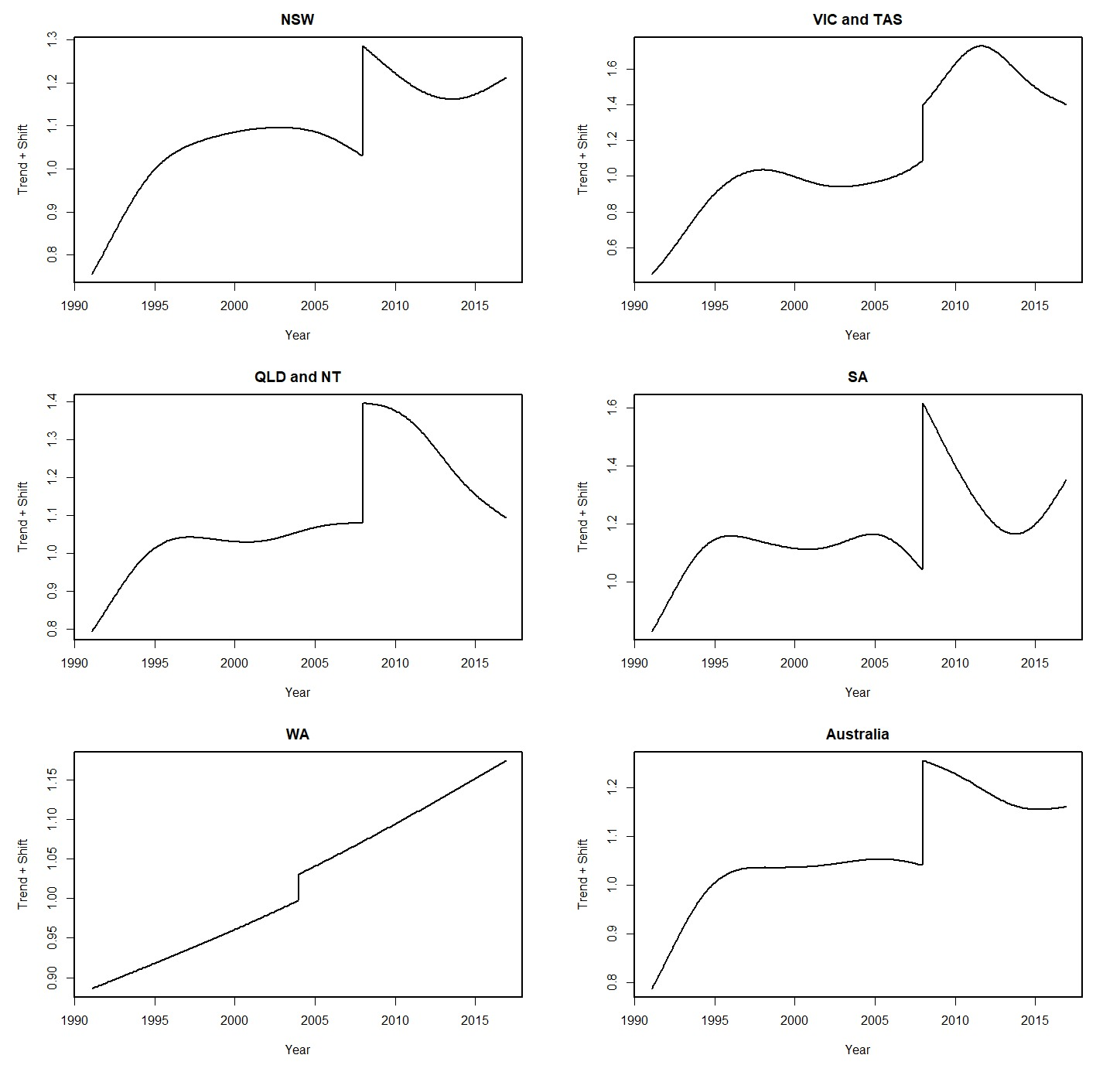
**

Figure S2: Estimated trend + shift components in % estimated from the model for monthly pertussis notifications from January 1991 - December 2016 by age groups and for all ages (from upper left: <6 months, 6 months – 4 years, 5–9 years, 10–64 years, ≥65 years, and all ages). Note that the shift (i.e., changepoint) happened in mid-2008 for all groups.


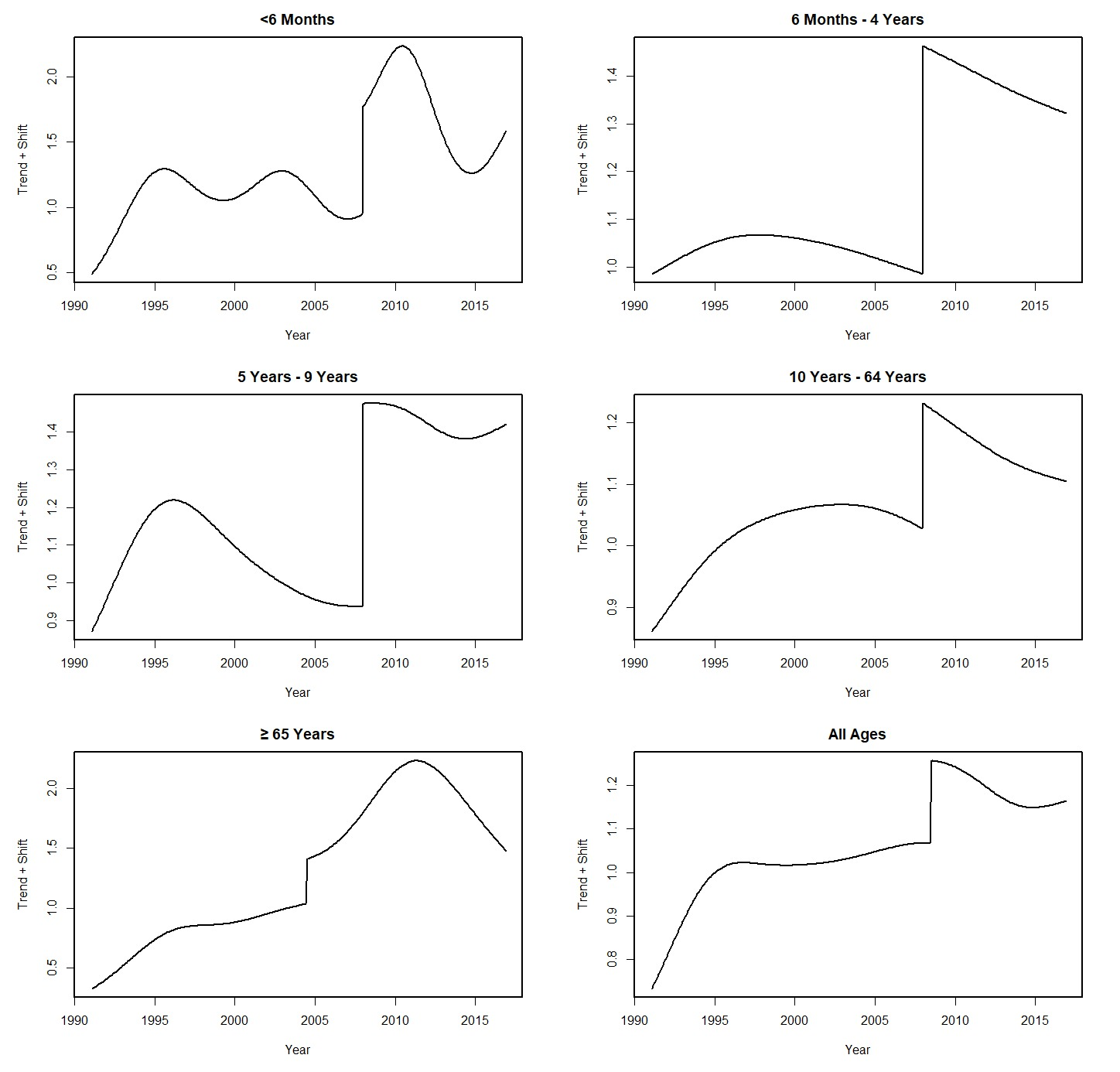


**Supplementary Material S3: Results from Sensitivity Analysis**

Table S1: Computed multiannual cycle lengths for each series based on entire data (middle column) and based on data prior to the identified changepoint (last column). Lengths were determined using visual inspection of periodograms, and the values presented are rounded up to a full month.

|  | Entire Series | Before Changepoint |
| --- | --- | --- |
| *Australia* | 3 years + 10 months | 3 years + 8 months |
|  |  |  |
| States/Territories |  |  |
| *New South Wales* | 3 years + 10 months | 3 years + 8 months |
| *Victoria and Tasmania* | 3 years | 3 years |
| *Queensland and Northern Territory* | 3 years + 10 months | 3 years + 8 months |
| *South Australia* | 4 years + 5 months | 4 years + 6 months |
| *Western Australia* | 3 years + 10 months | 4 years + 6 months |
|  |  |  |
| Age Groups |  |  |
| *<6 Months* | 3 years + 10 months | 3 years + 8 months |
| *6 Months – 4 Years* | 3 years + 10 months | 3 years + 8 months |
| *5 Years – 9 Years* | 3 years + 10 months | 3 years + 8 months |
| *10 Years – 64 Years* | 3 years + 10 months | 3 years + 8 months |
| *≥65 Years* | 5 years + 4 months | 4 years + 6 months |

Table S2: Summary of key seasonal signatures of monthly pertussis notifications from January 1991 - December 2016 for all of Australia and for each subgroup (by states and territories and by age groups) estimated from models without AR(1) component. Numbers inside ( ) are the estimated 95% confidence intervals.

|  | Peak Month | Yearly  Amplitude | Multiannual Amplitude |
| --- | --- | --- | --- |
| *Australia* | January  (12.94, 1.80) | 1.30  (1.23, 1.39) | 1.31  (1.23, 1.40) |
|  |  |  |  |
| States/Territories |  |  |  |
| *New South Wales* | January  (12.73, 1.95) | 1.29  (1,19, 1.41) | 1.24  (1.14, 1.35) |
| *Victoria and Tasmania* | January  (1.29, 2.35) | 1.18  (1.26, 1.34) | 1.29  (1.21, 1.38) |
| *Queensland and Northern Territory* | February  (1.52, 2.58) | 1.19  (1.27, 1.36) | 1.40  (1.30, 1.50) |
| *South Australia* | December  (11.89, 1.04) | 1.29  (1.26, 1.54) | 1.87  (1.68, 2.07) |
| *Western Australia* | January  (1.76, 2.33) | 1.37  (1.24, 1.51) | 1.37  (1.24, 1.51) |
|  |  |  |  |
| Age Groups |  |  |  |
| *<6 Months* | February  (1.73, 2.59) | 1.36  (1.27, 1.45) | 1.35  (1.25, 1.45) |
| *6 Months – 4 Years* | February  (2.53, 3.46) | 1.35  (1.25, 1.45) | 1.29  (1.20, 1.39) |
| *5 Years – 9 Years* | January  (12.96, 1.73) | 1.48  (1.37, 1.60) | 1.29  (1.18, 1.40) |
| *10 Years – 64 Years* | January  (12.76, 1.60) | 1.30  (1.23, 1.38) | 1.33  (1.26, 1.42) |
| *≥65 Years* | December  (12.37, 1.35) | 1.28  (1.20, 1.36) | 1.29  (1.21, 1.38) |

Figure S3: Actual (blue $-$) and fitted (red $-$) monthly pertussis notifications from January 1991 – December 2016 by states and territories and for all of Australia from models without AR(1) component (from upper left: New South Wales (NSW), Victoria and Tasmania (VIC and TAS), Queensland and Northern Territory (QLD and NT), South Australia (SA), Western Australia (WA), and Australia).


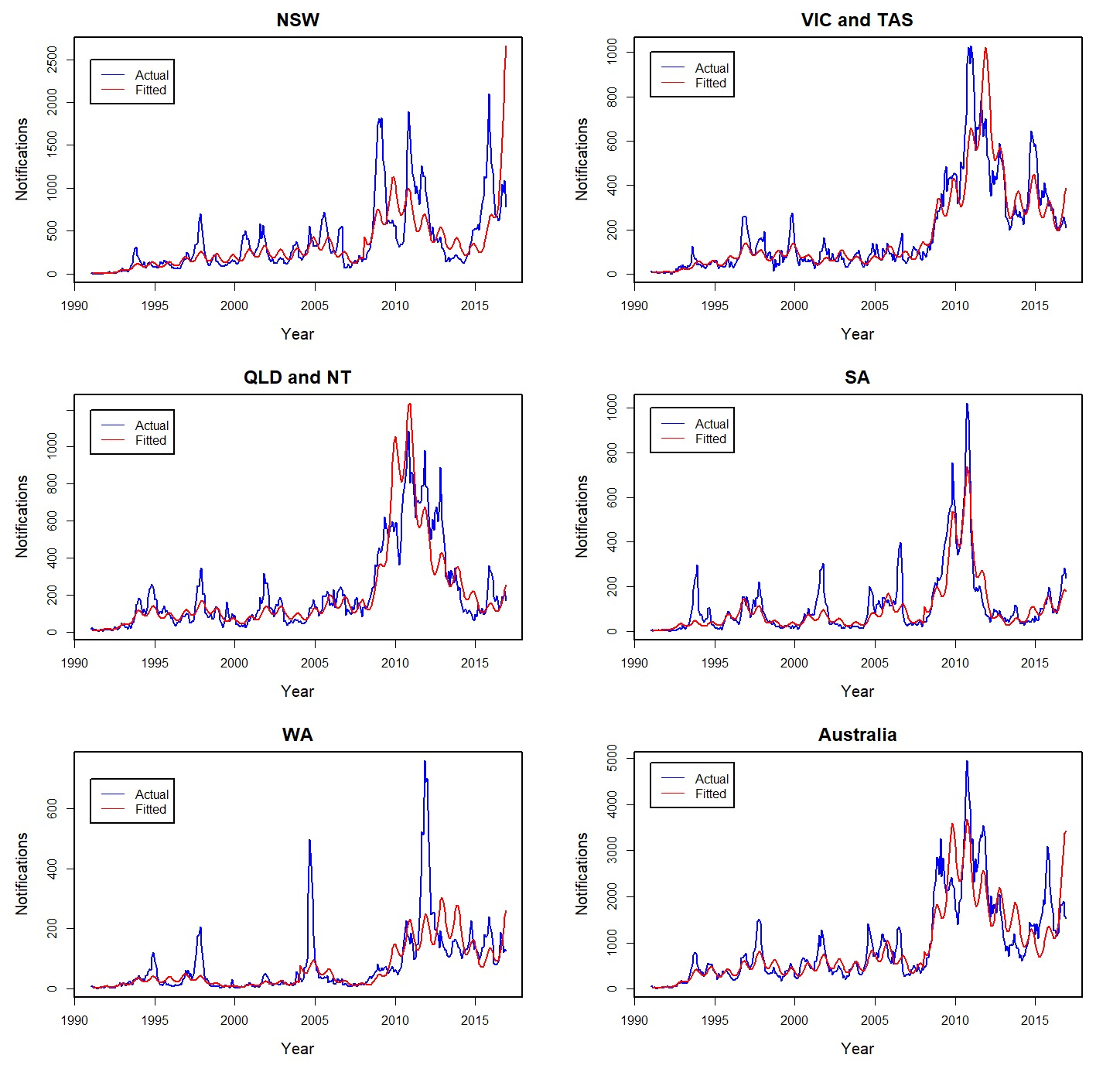


Figure S4: Actual (blue $-$) and fitted (red $-$) monthly pertussis notifications from January 1991 - December 2016 2016 by age groups and for all ages from models without AR(1) component (from upper left: <6 months, 6 months – 4 years, 5–9 years, 10–64 years, ≥65 years, and all ages).


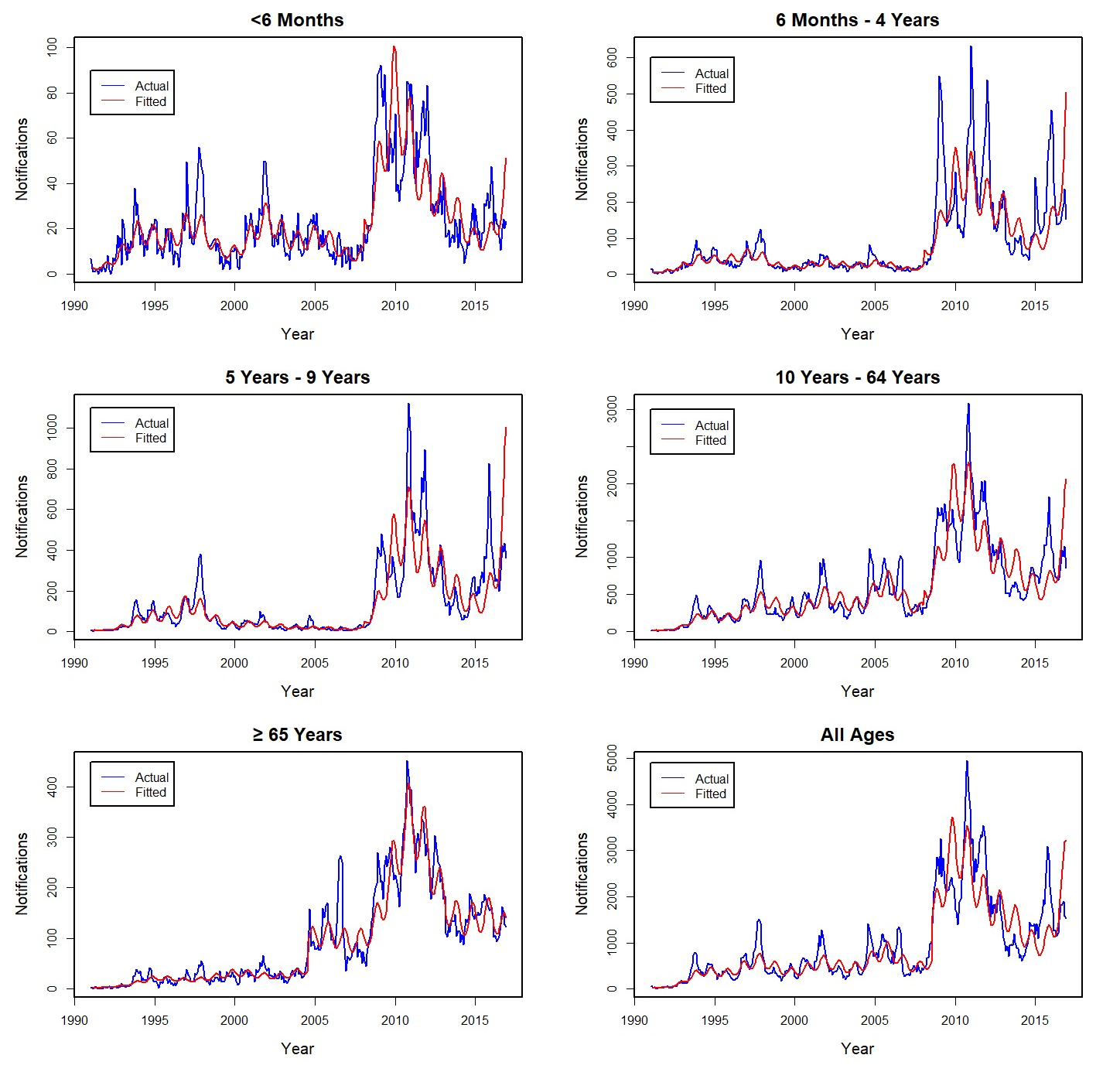

Supplement: Supplementary file 1 [file S0950268818003680sup001.docx]
